# Two Different Copy Number Variations of the SOX5 and SOX8 Genes in Yak and Their Association with Growth Traits

**DOI:** 10.3390/ani12121587

**Published:** 2022-06-20

**Authors:** Zhilong Zhang, Min Chu, Qi Bao, Pengjia Bao, Xian Guo, Chunnian Liang, Ping Yan

**Affiliations:** 1 Key Laboratory of Yak Breeding Engineering Gansu Province, Lanzhou Institute of Husbandry and Pharmaceutical Sciences, Chinese Academy of Agricultural Sciences, Lanzhou 730050, China; zhzhlo16@163.com (Z.Z.); chumin@caas.cn (M.C.); baoqinetwon@163.com (Q.B.); baopengjia@caas.cn (P.B.); guoxian@caas.cn (X.G.); 2 Key Laboratory of Animal Genetics and Breeding on Tibetan Plateau, Ministry of Agriculture and Rural Affairs, Lanzhou 730050, China

**Keywords:** copy number variation (CNV), SOX5, SOX8, growth traits, yak

## Abstract

**Simple Summary:**

As a domestic animal living in the Qinghai-Tibet Plateau at high altitudes from 2000 to 5000 m, the growth rate of the yak is slow under extremely harsh natural environmental conditions, such as high altitude, low temperatures, and low oxygen. Compared with conventional selection, using molecular markers in breeding to improve yak growth traits is more efficient. Many studies have indicated that CNV mutations can significantly affect the phenotypic traits of livestock and poultry. This study explored the association between the growth traits and CNVs of SOX5 and SOX8 in 326 Ashidan yaks. Our results showed that CNVs and the CNV combination of SOX5 and SOX8 were significantly associated with withers height and chest girth (*p* < 0.05), suggesting these mutations could be new markers for the selection of yak growth traits.

**Abstract:**

Copy number variation (CNV) is a structural variant with significant impact on genetic diversity. CNV has been widely used in breeding for growth traits, meat production or quality, and coat color. SRY-like box genes (SOXs) are a class of transcription factors that play a regulatory role in cell fate specification and differentiation. SOX5 and SOX8 belong to subgroups D and E of the SOXs, respectively. Previous studies have shown that SOX5 and SOX8 are essential in the development of bones. In this study, we explored the association between the growth traits and CNVs of SOX5 and SOX8 in 326 Ashidan yaks and detected mRNA expression levels in different tissues. Our results illustrated that CNVs of SOX5 and SOX8 were significantly associated with withers height at 18 months of age and chest girth at 30 months of age (*p* < 0.05). The CNV combination of SOX5 and SOX8 was significantly associated with withers height at 18 months of age (*p* < 0.01). SOX5 expression in the lung was significantly higher than in the heart, spleen, kidney, and muscle (*p* < 0.05). SOX8 expression in the lung was significantly higher than in the liver and muscle (*p* < 0.05). Our results provide evidence that the CNVs of SOX5 and SOX8 genes could be used as new markers for the selection of yak growth traits.

## 1. Introduction

The yak is a rare Chinese livestock species mainly living in the Qinghai-Tibet Plateau at high altitudes from 2000 to 5000 m [1]. Extremely harsh environmental conditions, including high altitude, low temperatures, and strong ultraviolet radiation enable yaks to have better oxygen transport, high energy metabolism, and a high capacity of the lungs and heart to ensure normal development [2]. At the same time, as the main local livestock species, the yak not only provides the local herders with daily necessities such as meat, milk and fur, but also is the main source of income for herders [3]. Research on the animal genetics and breeding of the yak, on the one hand, is helpful for the development of local animal husbandry and, on the other hand, is of great value to study the adaptation of animals and humans to the plateau. The Ashidan yak is the first hornless yak in the world that has the characteristics of a high meat production rate and a mild temperament. These excellent characteristics make it possible to carry out large-scale and intensive yak farming in the Qinghai-Tibet Plateau, which promotes protection of the ecological environment and the development of the economy in the region. In order to further promote the genetic improvement of the Ashidan yak and the development of animal husbandry, it is very important to use modern molecular marker technology to perform selective breeding for growth traits. As a molecular marker method, CNV (copy number variation) has significant effects on the growth traits of livestock and poultry, so it is a scientific and feasible method for performing selective breeding of the Ashidan yak.

With the development of animal genetics and breeding technology, molecular markers such as SNP (single-nucleotide polymorphism), indel (insertion/deletion,) and SVs (structural variants) have been widely used in the improvement and selection of economic traits in livestock and poultry [4,5,6]. The characteristics of the richness in eukaryotic genomes, high level of polymorphism, and excellent reproducibility in SNP make it one of the most popular and commonly used molecular marker technologies [7]. Unlike SNP with single-nucleotide mutations, an indel is defined as an insertion or deletion of segments less than 50 bp and can be detected by PCR amplification. These features make Indel more convenient and more efficient for detection [8]. To date, many studies have revealed a correlation between indel variation and the growth traits, reproductive performance, and milk traits of livestock and poultry [9,10,11,12]. CNV as a format of structural variants has more significant impact on genetic diversity than SNP and indel [13]. Abnormally sized fragments of CNV are more than 50 bp and are usually caused by genomic deletion, insertion, recombination, and complicated variants of numerous chromosomal loci [14,15]. Such variation in genome structure can affect phenotypic changes through multiple mechanisms, including changes in gene dosage, expression regulation, and exposure of recessive alleles [16]. Noncoding CNV variation in *cis*-regulatory element regions has been shown to have dramatic impact on phenotype during tissue development-specific regulation [17]. Through the study of a large number of recessive disease genes, it has been found that the formation of allelic carrier architecture and recessive disease-causing variations are related to CNV [18]. With the rapid development of genomics, biotechnology, and bioinformatics numerous CNVs have been discovered and applied to the markers of individual phenotypic traits and the interpretation of specific biological process mechanisms [19]. Whole-genome sequencing of 75 individuals, including eight cattle breeds, found 11,486 CNVRs (copy number variable regions), and a set of CNVRs had a significant effect on coat color and meat production or quality [20]. Similarly, the genome-wide CNV detection of 29 Chinese domesticated bulls was performed, and a total of 486 CNVRs were detected, accounting for 2.45% of the genome. Further examination of these CNVRs revealed that CNVR22 and CNVR310 were associated with body measurements [21]. Whole-genome CNV detection and association analysis in 2230 Nellore cattle found there were 231 CNVs in the whole genome, of which 17 CNVs were closely related to seven body traits [22]. Association analysis between CLCN2 and growth traits in Yunling cattle revealed the combination of two CNV mutations was significantly associated with cannon circumference [23]. These studies show that CNV is an important class of molecular genetic markers which has enormous value in revealing the formation mechanisms of phenotypic traits.

SOX proteins are a family of transcription factors with a highly conserved high-mobility group (HMG) domain, and 20 members with different functions were initially found during vertebrate embryo development [24,25,26]. Sox5 belongs to the subgroup D of the SOX family proteins. Previous studies have shown that Sox5 cooperates with other SOX family members to regulate the expression of Col2a1, a gene related to chondrogenesis, and has a persistent role during chondrocyte development. Further research revealed that Sox5 mutations affected the process of chondrogenesis, leading to serious skeletal malformations [27]. Sox8 belongs to subgroup E of the SOX family proteins. Research has shown that Sox8 is expressed in skeletogenic mesenchyme, which can significantly downregulate the expression of the osteoblast differentiation transcription factor RUNX2, causing mice to exhibit severely impaired bone formation [28,29]. Based on the above research results, we can conclude that SOX5 and SOX8 genes are necessary in the formation and normal development of the bones. In addition, the study of CNVRs in the yak genome has identified CNVs present in yak populations, and the potential effect of these CNVs on economic traits needs further study in a large population [30]. CNV293 and CNV992, identified in yak genome CNVR detection, overlap with SOX5 and SOX8, and belong to a gene family. Performing association analysis between CNVs of SOX5 and SOX8 and growth traits is of great value for discovering the potential effect of CNV and the trait formation mechanisms of different genes in a gene family. Therefore, we selected the SOX5 and SOX8 genes as candidate genes, performing quantitative real-time PCR (qPCR) to investigate the interrelations between them and growth traits, and we compared gene expression levels in different tissues. This could provide a theoretical reference for SOX5 and SOX8 as candidate genes for yak growth traits.

## 2. Materials and Methods

### 2.1. Ethics Statement

Growth trait measurements and blood and tissue sample collection were performed in strict accordance with the instructions for the Care and Use of Laboratory Animals. All experimental procedures were approved by the Animal Administration and Ethics Committee of Lanzhou Institute of Husbandry and Pharmaceutical Sciences of Chinese Academy of Agricultural Sciences (No. LIHPS-CAAS- 2017-115).

### 2.2. Sample Collection and Growth Trait Measurements

We randomly selected 326 female Ashidan yaks with similar health and nutritional levels from the Datong yak farm located in Qinghai Province of China. Four growth traits, body weight (BW), withers height (WH), body length (BL), and chest girth (CG), were measured at 6, 12, 18, and 30 months, respectively. The method of growth trait measurements was the reference standard used by Gilbert et al. [31]. Tissue samples (heart, spleen, liver, lung, kidney, muscle) of 3 female Ashidan yaks at 18 months of age were collected to perform gene expression studies.

### 2.3. Genomic DNA Extraction and RNA Isolation

Total RNA of tissue samples and genomic DNA of blood were extracted using TRIZOL reagent and TIANamp Blood DNA Kit (Tiangen Biotech, Beijing, China), respectively. The quality of RNA and DNA was detected by Thermo Scientific NanoDrop 2000c (ThermoFisher Scientific Inc., Waltham, MA, USA) and 1.2% agarose gels electrophoresis. Reverse transcription of RNA was performed with PrimeScript™ 1st Strand cDNA Synthesis Kit (TaKaRa Bio Inc., Dalian, China).

### 2.4. Candidate Gene information

**Figure 1:**
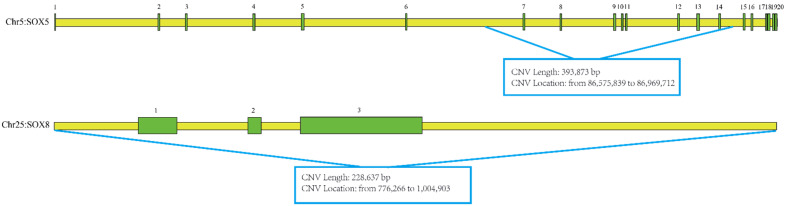
Information on CNVs of SOX5 and SOX8 genes. The numbers from 1 to 20 denote the exons.

The CNV (chr5: 86,575,839 to 86,969,712) of SOX5 (AC_000162.1) is located in intron 6–14. The CNV (chr25: 776,266 to 1,004,903) contains the entire gene sequence of SOX8 (AC_000182.1) (Figure 1).

### 2.5. Primer Design

Primers for detection of CNV and mRNA levels were designed using Primer Premier 5 software (Table 1). The optimum temperature of primers was determined by 1.5% agarose gel electrophoresis detection of PCR products under different temperature gradients.

### 2.6. Quantitative PCR Analysis

The basic transcription factor 3 (BTF3) and glyceraldehyde-3-phosphate dehydrogenase (GAPDH) were selected as reference genes to perform CNV detection and mRNA-level examination [32]. The LightCycler^®^ 96 Instrument (Roche, Basel, Switzerland) was used in our research. The 20 μL reaction mixtures contained 10 μL SYBR^®^ Green Premix Pro Taq HS qPCR Kit (Accurate Biology, Hunan, China), 6.8 μL ddH_2_O, 0.8 μL of upstream and downstream primers, and 1.6 μL of DNA/cDNA. The qPCR conditions were 60 s at 95 °C, and then 45 cycles of 10 s at 95 °C, and then 30 s at 60 °C. Subsequently, the melting curve was 30 s at 95 °C, and 0.02 s at 55 °C, and then 0.5 °C per cycle to 95 °C. Analysis of each DNA and RNA sample was replicated three times, and the experimental results were represented by mean values ± standard error (SE).

### 2.7. Statistical Analysis

CNV determination of SOX5 and SOX8 genes was based on the formula 2 × 2^−∆∆ct^ [23,33]. Results were divided into deletion, normal, and duplication based on copy numbers less than 2, equal to 2, and greater than 2. Furthermore, the association analysis for SOX5 and SOX8 CNVs and growth traits of Ashidan yaks was carried out by one-way analysis of variance (ANOVA). The model for evaluating the CNV effects on phenotypic traits was Y_ij_ = μ + CNV_i_ + e_ij_, where Y_ij_ is the observation of the growth traits; CNV_i_ is the effect of SOX5/SOX8-CNV type; μ refers to the overall mean of each trait; and e_ij_ refers to the random residual error. Additionally, the method of least-significant differences (LSD) was used to assess differences between means. The software used for data analysis was IBM SPSS Statistics software (Version 19, IBM, New York, NY, USA).

## 3. Results

### 3.1. Association Analysis between CNV and Growth Traits

For the purpose of exploring the association between SOX5 and SOX8 gene CNVs and Ashidan yak growth traits, we divided all individuals into three types: deletion, normal, and duplication. At the same time, the CNV combination type was determined according to the SOX5 and SOX8 gene CNV types of all individuals. The results revealed that SOX5-CNV was significantly associated with withers height at 18 months of age (*p* < 0.01). The withers height of the duplication type was significantly lower than that of the deletion and normal types (*p* < 0.05) (Table 2). SOX8-CNV had a significant effect on chest girth at 30 months of age (*p* < 0.05). The chest girth of the normal type was significantly lower than that of the deletion and duplication types (*p* < 0.05). No significance was detected between deletion and duplication types (*p* > 0.05) (Table 3). Furthermore, the CNV combination of SOX5 and SOX8 was significantly associated with withers height at 18 months of age (*p* < 0.01). The withers height of the deletion/deletion type was significantly higher than that of the duplication/normal and duplication/duplication types (*p* < 0.05). Means of the deletion/duplication, normal/deletion, and normal/duplication types were significantly higher than those for the duplication/duplication type (*p* < 0.05). The means of the deletion/duplication, normal/deletion, and normal/duplication types were not significantly different from each other (*p* > 0.05) (Table 4).

### 3.2. Gene Expression Profiling of SOX5 and SOX8

**Figure 2:**
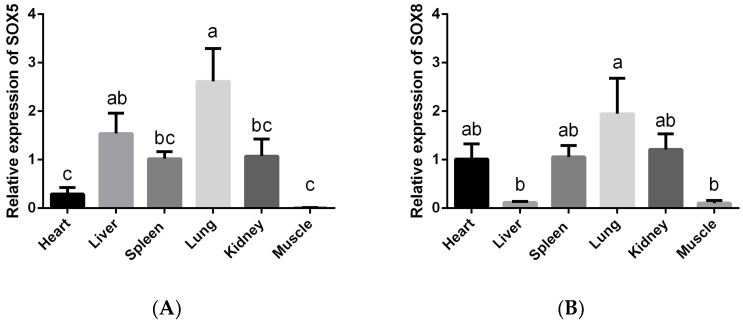
Gene expression of SOX5 and SOX8 in different tissues. (**A**) SOX5 expression in different tissues, and (**B**) SOX8 expression in different tissues. a, b, and c denote significant at *p* < 0.05.

To further illustrate the role of SOX5 and SOX8 genes in the growth and development of the Ashidan yak, mRNA expression levels in different tissues were analyzed in our study. The expression level of SOX5 in lung was significantly higher than that in heart, spleen, kidney, and muscle (*p* < 0.05). The expression in liver was significantly higher than in heart and muscle (*p* < 0.05) (Figure 2A). The SOX8 expression level in lung was significantly higher than that in liver and muscle (*p* < 0.05). The expression in heart, spleen, and kidney were not significantly different from each other (*p* > 0.05) (Figure 2B).

## 4. Discussion

In our study, SOX5 and SOX8 were selected as candidate genes, and the associations between CNV mutations and BW, WH, BL, and CG of 326 Ashidan yaks at four stages were analyzed. The SOX5-CNV was significantly associated with withers height at 18 months of age, and the withers height of the duplication type was significantly lower than that of the deletion and normal types. The SOX8-CNV had a significant effect on chest girth at 30 months of age, and the chest girth of the normal type was significantly lower than that of the deletion and duplication types. These findings illustrate that CNVs of SOX5 and SOX8 genes could be new molecular markers for the selection of yak growth traits. Furthermore, we carried out association analysis between CNV combinations of SOX5 and SOX8 and growth traits. The results showed that the CNV combination was significantly associated with 18-month withers height. It is known that the formation of quantitative traits is the result of the contribution and interaction of numerous genes. Therefore, we propose that the SOX5 and SOX8 genes work together on withers height during yak growth and development. To sum up, selection for growth in yaks may be enhanced by combining the CNV mutations of multiple genes. SOXs are a class of transcription factors widely expressed during development. A study found that SOX proteins regulate the Wnt pathway by interacting with β-catenin and/or TCF/LEF transcription factors [34]. Research on the roles of SOX transcription factors in skeletogenesis found that different SOX family members play different roles, and there were also synergistic effects. Mutations in these genes lead to skeletal dysmorphism [35]. SOX5 and SOX8 belong to the SOX family proteins of subgroups D and E, respectively. Allen et al. have pointed out that the haploinsufficiency of SOX5 at 12p12.1 is one of the reasons for individuals exhibiting developmental delays and mild dysmorphic features [36]. Similarly, a study using whole-exome sequencing technology found that a loss-of-function variant in SOX5 caused individuals to exhibit moderate developmental delay, mildly dysmorphic features, and scoliosis [37]. Katrina et al. found that SOX8 was expressed in a variety of tissues and involved in key developmental processes by studying the development of chick embryogenesis [38]. During mesodermal limb chondrogenesis, Sox8 was proved to be the most precocious marker [39]. It is worth pointing out that the abnormal expression of Sox8 leads to severely impaired bone formation in mice [29]. In our research, we found that the CNVs of SOX5 and SOX8 had significant effects on withers height and chest girth. Bone development has a direct impact on withers height and chest girth. Therefore, we infer that SOX5 and SOX8 may be necessary in the development of yak bones, and the CNV of the genes could be utilized as a new marker for the selective breeding of withers height and chest girth. It is interesting that the expression profiling of SOX5 and SOX8 in different tissues of yak showed the highest expression in the lung. The yak is a domestic animal living in the Qinghai-Tibet Plateau under extremely harsh natural environmental conditions, such as high altitude and low oxygen. As a central functional organ in the respiratory system, the lung of the yak has a larger pulmonary alveolar area and thinner blood–air barrier and alveolar septum than lowland cattle [40]. These characteristics allow the yak lung to have higher oxygen transport and carrying capacity, which are essential for yak adaptation. We speculate that the high expression of SOX5 and SOX8 in the lung may play a role in the adaptation of the yak to the plateau.

In this study, we explored the association between the growth traits and CNVs of SOX5 and SOX8 in Ashidan yaks and measured gene expression in different tissues. The results demonstrated that the combination of SOX5 and SOX8 CNVs was significantly associated with withers height. In addition, mRNA expression of the two genes was significantly higher in the lung than in other tissues. Therefore, we speculated that combining the CNV mutations of multiple genes would enhance selection for growth traits and that high expression of SOX5 and SOX8 in the lung would be essential for the adaptation of yaks.

## 5. Conclusions

This is the first time that two gene CNV mutations in one gene family were detected simultaneously in yaks. The SOX5-CNV and the combination of SOX5 and SOX8 CNVs were significantly associated with withers height at 18 months of age. Therefore, combining multiple molecular markers may be helpful in the selective breeding of yaks.

## Figures and Tables

**Table 1:** Primer information.

Level	Gene	Primer Pair Sequences ^1^ (5′-3′)	Amplicon Length (bp)	Tm (°C)
DNA	BTF3	F: AACCAGGAGAAACTCGCCAA	166	63
		R: TTCGGTGAAATGCCCTCTCG		
	SOX5	F: GCTTCCCAGTTCGCTTAG	104	55.6
		R: TTTCTGCCTTGGATGCTC		
	SOX8	F: CCTTGGGTCACTCGGGTTG	141	63
		R: GCGGCTCGGATTCTTTCG		
mRNA	GAPDH	F: CCACGAGAAGTATAACAACACC	120	56.1
		R: GTCATAAGTCCCTCCACGAT		
	SOX5	F: AAGAAACTGGCTGCGTCTCA	168	56.1
		R: TAATGGCGGCAGTTGACCTT		
	SOX8	F: CCGCACATCAAGACGGAGCA	213	64
		R: TGACGGGTAGCCAGGGAACG		

^1^ F: forward primer; R: reverse primer.

**Table 2:** Association analysis between SOX5-CNV and growth traits.

Age	Growth Trait ^1^	CNV Type ^2^ (Mean ± SE)			*p*-Value ^3^
		Deletion (56)	Normal (123)	Duplication (147)	
6 months	BW (kg)	82.89 ± 1.39	84.96 ± 0.94	84.33 ± 0.86	0.469
	WH (cm)	94.75 ± 0.71	94.72 ± 0.48	94.00 ± 0.44	0.468
	BL (cm)	92.57 ± 0.98	92.30 ± 0.66	91.33 ± 0.60	0.418
	CG (cm)	123.13 ± 1.05	124.02 ± 0.71	124.47 ± 0.65	0.550
12 months	BW (kg)	81.24 ± 1.45	83.58 ± 0.99	82.72 ± 0.89	0.410
	WH (cm)	89.87 ± 0.57	90.62 ± 0.39	90.58 ± 0.35	0.518
	BL (cm)	95.07 ± 0.68	95.82 ± 0.46	96.30 ± 0.42	0.297
	CG (cm)	116.42 ± 0.68	117.34 ± 0.46	117.38 ±.042	0.449
18 months	BW (kg)	119.60 ± 1.95	122.70 ± 1.33	123.36 ± 1.29	0.264
	WH (cm)	103.77 ± 0.79 ^a^	103.44 ± 0.55 ^a^	100.03 ± 0.50 ^b^	<0.01 **
	BL (cm)	102.32 ± 0.78	101.84 ± 0.55	101.55 ± 0.49	0.699
	CG (cm)	139.62 ± 1.38	139.28 ± 0.96	137.24 ± 0.87	0.183
30 months	BW (kg)	152.95 ± 2.43	155.60 ± 1.64	156.41 ± 1.49	0.479
	WH (cm)	100.33 ± 0.79	99.77 ± 0.51	99.37 ± 0.47	0.558
	BL (cm)	112.31 ± 0.91	112.81 ± 0.59	113.42 ± 0.54	0.525
	CG (cm)	147.03 ± 1.34	146.32 ± 0.87	148.03 ± 0.81	0.352

^1^ BW (body weight); WH (withers height); BL (body length); CG (chest girth). ^2, a^ and ^b^ denote significance at *p* < 0.05. ^3,^ ** denotes significance at *p* < 0.01.

**Table 3:** Association analysis between SOX8-CNV and growth traits.

Age	Growth Trait	CNV Type ^1^ (Mean ± SE)			*p*-Value ^2^
		Deletion (109)	Normal (100)	Duplication (117)	
6 months	BW (kg)	83.24 ± 0.99	84.83 ± 1.04	84.90 ± 0.97	0.411
	WH (cm)	94.44 ± 0.51	94.09 ± 0.53	94.62 ± 0.49	0.757
	BL (cm)	91.46 ± 0.70	92.98 ± 0.73	91.41 ± 0.67	0.211
	CG (cm)	123.73 ± 0.75	124.25 ± 0.79	124.22 ± 0.73	0.863
12 months	BW (kg)	82.27 ± 1.04	83.62 ± 1.09	82.56 ± 1.00	0.646
	WH (cm)	90.24 ± 0.41	90.73 ± 0.43	90.46 ± 0.40	0.717
	BL (cm)	95.40 ± 0.49	96.23 ± 0.51	96.10 ± 0.47	0.437
	CG (cm)	117.02 ± 0.49	117.35 ± 0.51	117.24 ± 0.47	0.888
18 months	BW (kg)	122.94 ± 1.43	122.27 ± 1.52	122.01 ± 1.42	0.893
	WH (cm)	102.76 ± 0.61	102.05 ± 0.62	101.13 ± 0.58	0.154
	BL (cm)	102.21 ± 0.58	101.76 ± 0.59	101.43 ± 0.55	0.626
	CG (cm)	138.83 ± 1.03	138.62 ± 1.05	137.87 ± 0.98	0.775
30 months	BW (kg)	153.95 ± 1.76	157.33 ± 1.75	155.27 ± 1.70	0.391
	WH (cm)	100.47 ± 0.56	99.25 ± 0.55	99.35 ± 0.53	0.221
	BL (cm)	113.36 ± 0.64	112.20 ± 0.63	113.45 ± 0.62	0.290
	CG (cm)	147.99 ± 0.96 ^a^	145.18 ± 0.92 ^b^	148.45 ± 0.91 ^a^	0.027 *

^1, a^ and ^b^ denote significance at *p* < 0.05. ^2,^ * denotes significance at *p* < 0.05.

**Table 4:** Association analysis between CNV combination of SOX5 and SOX8 and growth traits.

Age	CNV Combination Type	Growth Trait ^1^ (Mean ± SE)			
		BW (kg)	WH (cm)	BL (cm)	CG (cm)
6 months	Deletion/Deletion (26)	85.42 ± 2.21	95.38 ± 0.94	93.92 ± 1.23	124.31 ± 1.35
	Deletion/Normal (24)	79.54 ± 2.09	93.71 ± 1.08	92.42 ± 1.80	121.13 ± 1.50
	Deletion/Duplication (6)	85.33 ± 5.12	96.17 ± 2.61	87.33 ± 1.36	126.00 ± 2.63
	Normal/Deletion (41)	82.54 ± 1.47	94.44 ± 0.81	90.68 ± 1.18	122.49 ± 1.35
	Normal/Normal (41)	87.85 ± 1.42	95.02 ± 0.96	93.98 ± 1.20	125.39 ± 1.27
	Normal/Duplication (41)	84.55 ± 1.64	94.68 ± 0.82	92.24 ± 1.37	124.17 ± 1.25
	Duplication/Deletion (42)	82.57 ± 1.61	93.86 ± 0.72	90.69 ± 1.00	124.60 ± 1.15
	Duplication/Normal (35)	85.00 ± 1.85	93.26 ± 0.84	92.20 ± 1.32	125.06 ± 1.39
	Duplication/Duplication (70)	85.07 ± 1.27	94.46 ± 0.66	91.27 ± 0.71	124.10 ± 0.92
***p* value** ^2^		0.109	0.787	0.216	0.501
12 months	Deletion/Deletion (26)	81.68 ± 2.58	90.08 ± 0.78	94.44 ± 1.08	117.08 ± 0.79
	Deletion/Normal (24)	79.17 ± 1.95	89.46 ± 0.81	95.33 ± 0.67	115.08 ± 0.96
	Deletion/Duplication (6)	87.33 ± 4.04	90.67 ± 1.76	96.67 ± 2.39	119.00 ± 1.84
	Normal/Deletion (41)	82.67 ± 1.45	89.73 ± 0.65	95.00 ± 0.68	116.33 ± 0.97
	Normal/Normal (41)	85.13 ± 1.59	91.63 ± 0.67	96.65 ± 1.10	118.65 ± 0.81
	Normal/Duplication (41)	82.95 ± 1.87	90.50 ± 0.70	95.80 ± 0.92	117.05 ± 0.65
	Duplication/Deletion (42)	82.24 ± 1.60	90.83 ± 0.68	96.36 ± 0.78	117.64 ± 0.75
	Duplication/Normal (35)	84.88 ± 1.87	90.57 ± 0.71	96.37 ± 0.77	117.43 ± 0.90
	Duplication/Duplication (70)	81.93 ± 1.32	90.42 ± 0.53	96.23 ± 0.50	117.20 ± 0.62
***p* value**		0.460	0.638	0.690	0.269
18 months	Deletion/Deletion (26)	122.86 ± 2.82	104.46 ± 1.42 ^a^	104.25 ± 1.32	141.00 ± 1.68
	Deletion/Normal (24)	116.20 ± 2.04	103.13 ± 1.17 ^a,b,c^	100.09 ± 1.11	137.91 ± 1.72
	Deletion/Duplication (6)	119.50 ± 7.54	103.50 ± 0.99 ^a,b^	103.17 ± 1.87	140.67 ± 4.06
	Normal/Deletion (41)	121.23 ± 2.88	103.56 ± 1.08 ^a,b^	101.12 ± 1.15	138.94 ± 2.38
	Normal/Normal (41)	125.09 ± 2.36	103.05 ± 0.90 ^a,b,c^	102.76 ± 0.91	139.66 ± 1.58
	Normal/Duplication (41)	121.68 ± 2.19	103.73 ± 0.66 ^a,b^	101.54 ± 0.90	139.22 ± 1.33
	Duplication/Deletion (42)	124.55 ± 2.58	101.03 ± 0.97 ^a,b,c^	101.90 ± 0.89	137.38 ± 1.57
	Duplication/Normal (35)	123.55 ± 2.37	100.09 ± 1.15 ^b,c^	101.78 ± 0.98	137.91 ± 1.49
	Duplication/Duplication (70)	122.46 ± 1.73	99.38 ± 0.74 ^c^	101.21 ± 0.64	136.81 ± 1.42
***p* value**		0.486	<0.01 **	0.320	0.770
30 months	Deletion/Deletion (26)	154.71 ± 3.67	101.20 ± 1.44	114.07 ± 1.31	149.60 ± 2.67
	Deletion/Normal (24)	149.68 ± 2.81	100.21 ± 1.19	110.53 ± 1.41	145.16 ± 1.33
	Deletion/Duplication (6)	159.40 ± 5.33	98.20 ± 0.73	113.80 ± 3.62	146.40 ± 1.94
	Normal/Deletion (41)	150.71 ± 2.98	99.79 ± 1.08	112.90 ± 1.05	146.90 ± 1.52
	Normal/Normal (41)	160.72 ± 2.85	99.15 ± 0.73	112.45 ± 1.07	144.50 ± 1.54
	Normal/Duplication (41)	154.70 ± 2.93	100.42 ± 0.80	113.10 ± 0.88	147.65 ± 1.63
	Duplication/Deletion (42)	156.30 ± 2.93	100.74 ± 1.09	113.45 ± 0.89	148.23 ± 1.53
	Duplication/Normal (35)	158.64 ± 2.79	98.72 ± 0.72	113.00 ± 1.21	145.93 ± 1.67
	Duplication/Duplication (70)	155.20 ± 2.20	98.80 ± 0.63	113.65 ± 0.78	149.19 ± 1.09
***p* value**		0.225	0.497	0.716	0.289

^1, a, b,^ and ^c^ denote significance at *p* < 0.05. ^2,^ ** denotes significance at *p* < 0.01.

## Data Availability

The study did not report any data.
